# Comparison of lauric acid and 12-hydroxylauric acid in the alleviation of drought stress in peach (*Prunus persica* (L.) Batsch)

**DOI:** 10.3389/fpls.2022.1025569

**Published:** 2022-10-21

**Authors:** Binbin Zhang, Hao Du, Maoxiang Sun, Xuelian Wu, Yanyan Li, Zhe Wang, Yuansong Xiao, Futian Peng

**Affiliations:** College of Horticulture Science and Engineering, Shandong Agricultural University, Taian, China

**Keywords:** *Prunus persica* (L.) Batsch, lauric acid, 12-hydroxylauric acid, drought stress, physiological indicators

## Abstract

Water shortage is a key factor that can restrict peach tree growth. Plants produce fatty acids and the fatty acid derivatives lauric acid (LA) and 12-hydroxylauric acid (LA-OH), which are involved in abiotic stress responses, but the underlying stress response mechanisms remain unclear. In this study, physiological examination revealed that in *Prunus persica* (L.) Batsch, pretreatment with 50 ppm LA-OH and LA reduced drought stress, efficiently maintained the leaf relative water content, and controlled the relative conductivity increase. Under drought stress, LA-OH and LA treatments prevented the degradation of photosynthetic pigments, increased the degree of leaf stomatal opening and enhanced the net photosynthetic rate. Compared with drought stress, LA-OH and LA treatment effectively increased the net photosynthetic rate by 204.55% and 115.91%, respectively, while increasing the Fv/Fm by 2.75% and 7.75%, respectively, but NPQ decreased by 7.67% and 37.54%, respectively. In addition, the level of reactive oxygen species increased under drought stress. The content of O_2_^-^ in LA-OH and LA treatment decreased by 12.91% and 11.24% compared to CK-D, respectively, and the content of H_2_O_2_ decreased by 13.73% and 19.94%, respectively. At the same time, the content of malondialdehyde (MDA) decreased by 55.56% and 58.48%, respectively. We believe that the main reason is that LA-OH and LA treatment have improved the activity of superoxide dismutase (SOD), peroxidase (POD), and catalase (CAT). The application of exogenous LA increased the levels of soluble sugars, soluble proteins, proline and free amino acids under drought stress, and maintained the osmotic balance of cells. Compared with CK-D treatment, it increased by 24.11%, 16.89%, 29.3% and 15.04%, respectively. At the same time, the application of exogenous LA-OH also obtained similar results. In conclusion, exogenous LA-OH and LA can alleviate the damage to peach seedlings caused by drought stress by enhancing the photosynthetic and antioxidant capacities, increasing the activities of protective enzymes and regulating the contents of osmotic regulators, but the molecular mechanism is still in need of further exploration.

## Highlights

- Exogenous LA-OH and LA enhanced the photosynthetic and fluorescent properties of drought-stressed leaves.
- LA-OH and LA treatment of *P. persica* could enhance osmoregulation and reduce oxidative stress.
- The equilibrium of reactive oxygen species might be better maintained under drought circumstances after LA-OH and LA pretreatment by increasing antioxidant enzyme activity.
- LA-OH and LA can improve the drought resistance of *P. persica*.

## Introduction

In recent years, with the exacerbation of global climate change and the destruction of the natural environment (Leng and Hall, 2019), drought stress has become common worldwide and is one of the most important factors affecting agricultural yields (Guo et al., 2014 **;** Han et al., 2022). Drought stress directly affects every stage of plant growth and development, from seed emergence to final seed setting, resulting in a series of physiological and biochemical reactions and gene expression changes throughout the plant (Bocchini et al., 2018). The detrimental effects of drought stress on crops include plant growth inhibition, decreased photosynthetic activity, excessive formation of reactive oxygen species, disruption of oxidative metabolism balance, increased damage to membrane lipid peroxidation, and even plant mortality (Caser et al., 2019; Liang et al., 2019; Lozano-Montaña et al., 2021; Sun et al., 2022). Studies have indicated that drought is the most problematic factor exacerbating anthropogenic climate change (Del Buono, 2021). At present, strategies such as breeding and genetic modification are being used for drought (Chukwuneme et al., 2020 **;** Maazou et al., 2016), including soil improvement and enhanced irrigation (Rauf et al., 2016). However, these methods are not very effective, because they not only consume a lot of time, but also have high labor and cost (Fahad et al., 2017 **;** Ullah et al., 2017). Thus, finding efficient techniques to alleviate plant stress is critical.

Using bioregulators or organic acids to increase plant growth and improve drought tolerance has been demonstrated to be an effective and environmentally beneficial strategy (Li et al., 2017 **;** Saidi et al., 2017 **;** Xa lxo and Keshavkant, 2019). Fatty acids are bioactive compounds that are classified as short-chain (SCFA; <C8:0), medium-chain (MCFA; C8: 0–14: 0), or long-chain (LCFA; >C16: ω3–9) based on the length of their carbon atom chains (Beermann et al., 2003 **;** Bocca et al., 2007). Lauric acid (LA), systematically designated dodecanoic acid, is a saturated fatty acid with similar properties to medium-chain fatty acids (Sandhya et al., 2016). Although the role of LA in the water stress response remains to be elucidated, it is noteworthy that the presence of LA in *in vitro* culture medium has been found to promote resistance to drought stress during the pre-domestication stage by maintaining leaf water concentration, stomatal closure and nonphotochemical energy dissipation, all of which can change plant metabolic rates (Medeiros et al., 2015). Plants grown in this lauric acid medium exhibited increased photosynthesis during the recovery period. Studies have shown that in loquat, LA may have an effect similar to that of capric acid and abscisic acid (ABA) on improving stomatal sensitivity and drought resistance in leaves (Gugliuzza et al., 2020). Through the study of fatty acid oxidase in organisms, it has been found that fatty acid oxidation products seem to have important biological activities (Funk, 2001 **;** Blée, 2002 **;** Noverr et al., 2003). Plants, like certain other organisms, create hydroxy fatty acids primarily through enzymatic processes mediated by cytochrome P450-dependent fatty acid hydroxylases (Benveniste et al., 2005). Different fatty acid hydroxylase metabolites are involved in different aspects of plant development and responses to biotic and abiotic stresses (Kandel et al., 2006). Researchers have cloned the fatty acid-metabolizing enzyme CYP94C1 of the cytochrome P450 family from *Arabidopsis thaliana*. When recombinant yeast microsomes were incubated with lauric acid (C12:0) for 15 min, a major metabolite was formed, identified as 12-hydroxylauric acid (Kandel et al., 2007). Studies have shown that synthetic medium-chain-hydroxy fatty acid metabolites can act as systemic immune elicitors (Kutschera et al., 2019). Although 12-hydroxylauric acid can be employed as an immune activator in plants, few studies have been conducted on its role in plant responses to biotic stress.

Peach (*Prunus persica* (L.) Batsch) is a popular commercial fresh fruit worldwide. Peach development is heavily reliant on an appropriate amount of water (Eldem et al., 2012). Plants respond to drought stress by activating several response mechanisms that govern various physiological processes while also influencing the biochemical processes related to plant morphology (Bielsa et al., 2021). To prevent water loss, leaf pigment levels and photosynthetic activity are decreased and growth is managed through moderating stomatal closure and osmotic management (Belin et al., 2009). Stomatal conductance (Gs), leaf relative water content (RWC), relative electrolytic leakage (REL), plant antioxidant system, and a variety of other metrics have been used to investigate this physiological response (Verslues et al., 2006; Golldack et al., 2014 **;** Lind et al., 2015). Drought stress can have a series of effects on cells, including an increase in the production of reactive oxygen species (ROS), destruction caused by membrane lipid peroxidation, inhibition of enzyme function, protein oxidation and changes in plant antioxidant systems (Li et al., 2011; Gao et al., 2020; Kaya et al., 2020). These characteristics provide strong theoretical support for plants’ capacity to endure drought stress.

To better understand plant stress responses, we induced natural drought stress in ‘Maotao’ peach cultivar seedlings (*Prunus persica* (L.) Batsch) in a greenhouse for this study. Over the course of the study, we investigated the effects of exogenous lauric acid (LA) and 12-hydroxylauric acid (LA-OH) on peach seedling responses to drought. Our findings indicate that exogenous LA and LA-OH can ameliorate the drought-associated oxidative damage and decrease in photosynthesis while also delaying drought-induced leaf senescence. These findings provide a strategy to boost agricultural production through enhanced drought tolerance.

## Materials and methods

### Experimental materials

This experiment was carried out at the Shandong Agricultural University experimental base, located in Tai’an city, Shandong Province, China (36°17′7459″N, 117°16′7712″E), in 2021. Uniform and plump peach seeds were selected and were soaked in a 400 ppm gibberellin solution for 24 hours before being placed in seedling trays. Once the peach seedlings reached approximately 5 cm in height, healthy seedlings of comparable size that were free of insect pests were selected for planting in pots. Each pot was a square shape (7 cm x 7 cm). The media in the pot was a 2:1 volume ratio blend of garden soil and vermiculite. Each pot of culture media weighed 200 g. The seedlings were maintained on a regular basis.

### Drought treatments

Peach seedlings that developed similarly were selected and split into three groups of 80 pots each. For three consecutive treatments, the seedlings were irrigated every five days with one of the following, depending on the group to which they were assigned: clean water, lauric acid (50 ppm, Sigma−Aldrich) or 12-hydroxylauric acid (50 ppm, Shanghai Macklin Biochemical Co., Ltd.). The reagent concentration was established by the results of earlier preparatory studies. We discovered that LA and LA-OH had no negative impact on the development of peach seedlings after the irrigation treatments. Then, each group of peach seedlings was split into two subgroups: one that received a regular watering treatment (CK, LA-OH, LA) and one that was exposed to natural drought conditions (CK-D, LA-OH-D, LA-D). There were 40 pots in each subgroup. Following a 14-day treatment period, leaves from all subgroups were washed carefully with tap water, frozen with liquid nitrogen, and then stored at -80°C until use.

### Calculation of the relative water content and relative electrolyte leakage of leaves

Three plants were chosen for each treatment, two leaves were removed from each plant and promptly placed in an aluminium box of known weight, and the fresh weight (Wf) was determined. The leaves were submerged in distilled water for approximately 1 hour before being removed and weighed to determine the saturated fresh weight (WT) of the sample. The dry weight (Wd) was then obtained by drying the leaves to a constant weight. The relative water content (RWC) was determined using the following formula (Bielsa et al., 2021): (Wf-Wd)/(Wt-Wd)×100%.

The relative electrolyte leakage (REL) was measured with reference to Gao et al. (2020) but with slight modifications. Ten tiny discs were punched from ripe leaves and placed in a 20 mL centrifuge tube with 10 mL of deionized water. After 4 hours at room temperature, a Raymag DDS-307 conductivity meter was used to test the solution conductivity, designated S1. At the same time, the conductivity of the deionized water, designated S0, was measured. The centrifuge tube was then placed in a 100°C water bath for 20 min. After cooling, the mixture was shaken well, and the conductivity, designated S2, was measured. To represent the relative permeability of the plasma membrane, the relative electrolyte leakage was calculated using the following formula: REL (%) = (S1-S0)/(S2-S0)×100%.

### Determination of gas exchange and photosynthetic parameters

On the 14th day of the treatment period, photosynthesis was monitored from 10:00 to 11:30 am. We measured the following parameters with the CIRAS-3 portable photosynthetic system (PP Systems, Massachusetts, USA): net photosynthetic rate (Pn), transpiration rate (Tr), stomatal conductance (Gs), intercellular CO_2_ concentration (Ci) and water use efficiency (WUE). The relative chlorophyll content (SPAD) was measured six times using a SPAD502 instrument, and the average value was calculated.

Samples (0.2 g) were taken from fresh, clean peach leaves and extraction was performed for 24 h in a 95% ethanol solution. We then used a Pharma-Spec UC-2450 ultraviolet spectrophotometer (Shimadzu, Kyoto, Japan) to measure the OD_665_, OD_649_, and OD_470_ of the extract. To calculate leaf chlorophyll and carotenoid levels, we used the methods of Li et al., 2021.

We used the IMAGING-PAM Chlorophyll Fluorescence System (Heinz Walz, Effeltrich, Germany) to measure the following parameters: minimal fluorescence (F0), maximal fluorescence (Fm), maximum quantum efficiency (Fv/Fm), actual quantum efficiency (ΦPSII), photochemical quenching (qP), nonphotochemical quenching (NPQ), and electron transport rate (ETR). To determine Fv/Fm, the peach leaves were dark-adapted in a dark clip adaptor for 30 min.

### Stomatal aperture assay

Three leaves from the same part of each plant were collected 14 days later to assess the stomata morphology and degree of opening. Colourless acrylic nail polish was applied evenly on the back of each collected peach leaf. After 5 min, tweezers were used to remove the nail polish after it had hardened. The piece treated with nail polish was then placed on a microscope slide and examined with a fluorescence microscope at 400× magnification (AXI0, Carl Zeiss, Germany). We chose three sites at random on each piece for imaging. To calculate and identify the degree of openness, stomatal aperture imprints were measured using ImageJ software (National Institutes of Health, Bethesda, Maryland, USA).

### Determination of osmotic regulatory substances

To calculate the total soluble sugar content (Li et al., 2021), 0.2 g of leaves was weighed, chopped and mixed. Then, 10 mL of distilled water was added and the leaf matter was placed in a boiling water bath for 30 min to create an extract, which was then filtered and diluted to a 25 mL volumetric flask. One millilitre of extract was mixed with 4 mL of 0.2% anthrone reagent, and the absorbance at 620 nm was measured.

The proline content in the leaves was then measured (Mehdizadeh et al., 2021). First, 5 mL of 3% sulfosalicylic acid was added to 0.5 g of leaf sample, and the sample was ground to prepare the homogenate, which was then boiled in a water bath for 10 min and cooled to room temperature. Then, 2 mL of supernatant was collected and added to 2 mL of glacial acetic acid and 3 mL of ninhydrin to generate a coloured liquid. The mixture was then heated in a boiling water bath for 40 min. In addition, 5 mL of toluene was added to the mixture for extraction, and the absorbance value of the toluene layer was measured using a spectrophotometer at 520 nm.

For enzyme extraction, 0.5 g fresh leaf sample were collected, and 0.1 mL of the extract was pipetted from the mixture and added to 5 mL of Coomassie Brilliant Blue G-250 reagent and mixed well. The mixture was allowed to rest for 2 min, and then the absorbance at 595 nm was recorded and the soluble protein concentration was assessed based on the standard curve (Xiao et al., 2020 **)**.

The method for measuring the total amount of free amino acids was based on Vardharajula et al., 2011, with slight modifications. Approximately 0.5 g of leaves was weighed, and 10% acetic acid was added to make a homogenate, which was then diluted to 100 mL. Then, 2 mL of sample extract was pipetted from the homogenate and mixed with 3 mL of ninhydrin reagent and 0.1% ascorbic acid aqueous solution. Next, the mixture was heated for 15 min in a boiling water bath and cooled to the colour of the solution, which was blue−violet. Then, 60% ethanol was added to 5 mL, and the absorbance was tested at 570 nm.

### Determination of leaf reactive oxygen species levels

We measured the hydrogen peroxide content in leaves using the methods of Xiao et al. (2020). Briefly, we placed cut-up leaf samples (0.1 g) in sterilized centrifuge tubes and then froze them with liquid nitrogen. After centrifugation at 60 rpm for 150 s, leaves were ground, frozen, and centrifuged again. The samples were then mixed with 1.5 mL of 0.1% trichloroacetic acid (TCA) and placed on ice. The samples were then centrifuged again at 12000 rpm for 15 min at 4°C. A 0.5 ml sample of supernatant was then collected and mixed with 0.5 mL of phosphate-buffered saline (PBS) and 1 mL of potassium iodide (KI). The resulting solution was shaken well and kept at 28 °C for 1 hour, after which we measured the absorbance at 390 nm.

We measured the O_2_^-^ content in leaves using the methods of Han et al. (2022). Briefly, we chopped up 1 g sampled peach leaves and added 3 mL of phosphate buffer (pH = 7.8). The samples were then placed in an ice bath and were ground and then centrifuged at 4000 × g for 15 min. The supernatant was collected and mixed with 0.1 mL of 10 m mol/L hydroxylamine hydrochloride solution and incubated at 25°C for 20 min. We then added 1 mL of 17 m mol/L p-aminobenzene sulfonic acid and 1 mL of 7 m mol/L a-naphthylamine solution and incubated the mixture at 25°C for 20 min. After that, we added an equal volume of chloroform to extract the pigment and centrifuged the mixture at 10000 r/min for 3 min. The pink extract was collected to measure the OD_530_.

### Measurements of leaf lipid peroxidation

Malondialdehyde (MDA), a biomarker of lipid peroxidation caused by oxidative stress, was measured using the method described by Zhang et al. (2016), with some modifications. Briefly, we mixed 0.20 g leaf samples with 2 mL of 0.1% trichloroacetic acid (TCA) and ground the samples to prepare the homogenate. Samples were then centrifuged at 4000 r/min for 10 min. We collected 1 mL of supernatant and mixed it with 4 mL of 20% TCA (containing 0.5% thiobarbituric acid [TBA]), placed the mixture in a boiling water bath for 60 min for reaction to occur, and then cooled the mixture quickly on ice at 4 °C. The absorbance OD values at 600, 532 and 450 nm were measured after centrifugation at 4000 rpm for 10 min. MDA content was expressed as U/g·min.

### Determination of leaf SOD, POD and CAT activities

The mixed sample (0.5 g) was weighed and ground in liquid nitrogen, and 4 mL of phosphate buffer was added at a concentration of 0.05 M pH 7.8 (0.3% EDTA with 0.1 mM, Triton-X100 and 4% polyvinylpyrrolidone) was ground fully into a centrifuge tube, with a 6 mL buffer flush applied twice at 4 °C and 12,000 r/min. After centrifugation for 20 min, the supernatant was collected and stored at 4°C until use.

Superoxide dismutase (SOD) activity was determined according to the method of **Zhang et al. (2016)**, with some modifications. The reaction mixture consisted of 1 μM nitro blue tetrazolium (NBT), 30 μM EDTA, 60 μM riboflavin, 14 mM methionine, 50 mM sodium phosphate buffer (pH 7.8), and 100 μL of crude enzyme extract. After the samples were evenly mixed, they were incubated in a water bath at 37°C for 30 min, and then the absorbance was read at 560 nm.

Peroxidase (POD) activity was determined according to the methods of Liang et al. (2018), with some modifications. One millilitre of crude enzyme extract was mixed with 1.0 mL of guaiacol, 1.0 mL of H_2_O_2_, and 2 mL of 0.2 mM phosphate buffer (pH 5.0) and heated at 34°C for 3 min. To start the reaction, H_2_O_2_ was added, and then the A470 value was measured as A1. The absorbance A2 was measured after 1 min and 30 s, and the POD activity was recorded in terms of activity per gram of fresh tissue.

Catalase (CAT) activity was determined according to the methods of Li et al. (2011), with slight modifications. Briefly, we added 0.2 mL of crude enzyme extract to 2.8 mL of 30 mM H_2_O_2_ and measured the absorbance at 240 nm after 5 s. Then, we determined the initial absorption value A1 and the absorption value A2 after 1 min. Activity was expressed in CAT enzyme activity units, for which one unit equals a change of 0.01 in the A240 value per gram of tissue per minute per millilitre of reaction solution.

### Statistical analysis

SPSS 17.0 (IBM, New York, USA) statistical analysis software was used to perform one-way ANOVA and Duncan’s multiple comparison test. Statistical significance, presented as lettered results in the tables below, was assumed at a level of 5% (P < 0.05). Data on all tables are presented as the means ± standard deviations (error bars).

## Results

### LA and LA-OH enhanced drought stress tolerance

**Figure 1:**
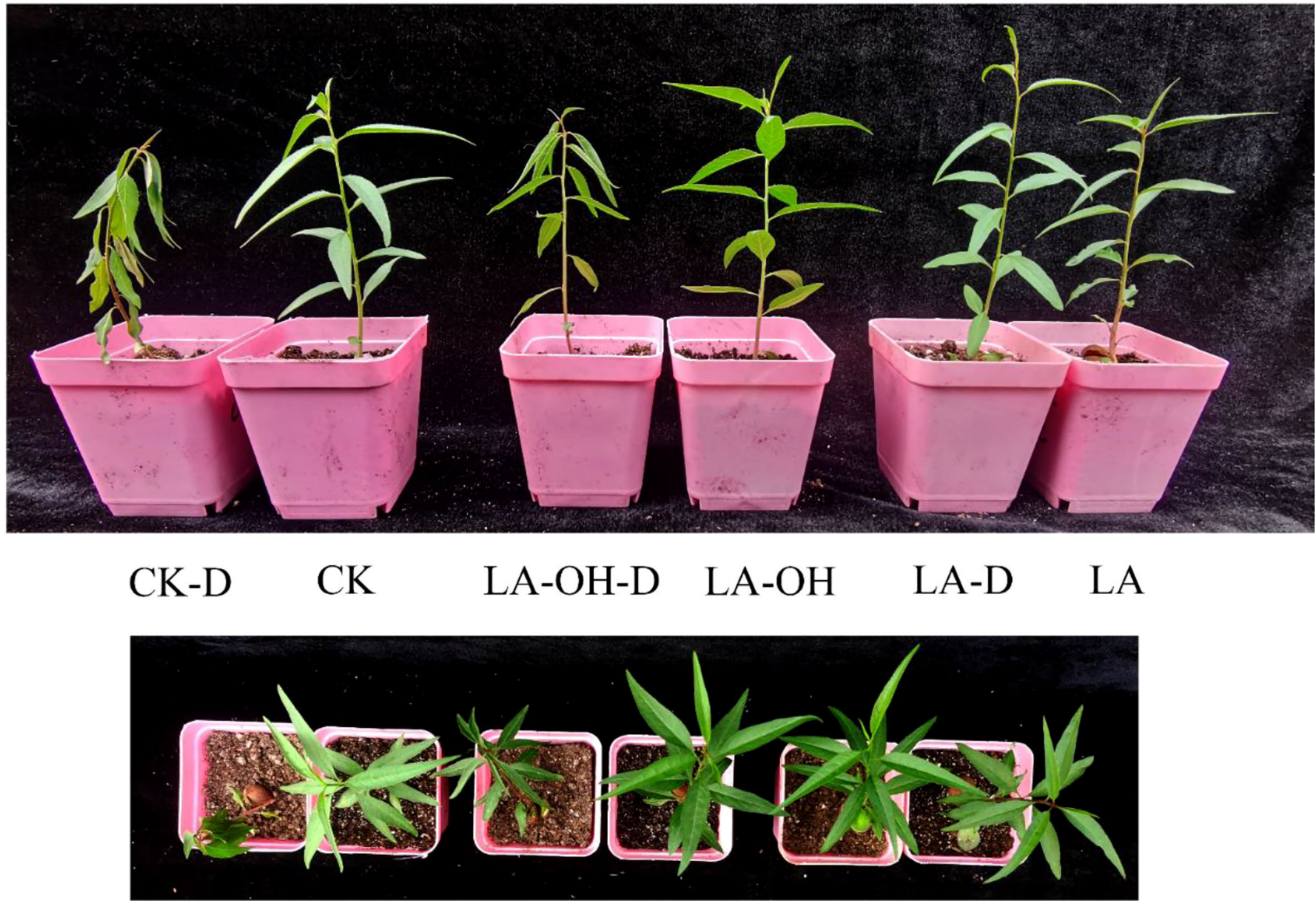
Drought tolerance of *P. persica* was increased by 12-Hydroxylauric acid and Lauric acid pretreatment. The detailed treatments of CK-D, CK, LA-OH-D, LA-OH, LA-D and LA are described in Materials and methods 2.2.

**Figure 2:**
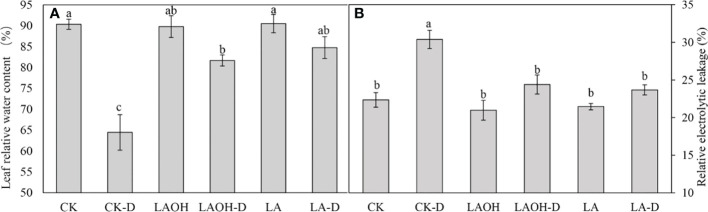
The effects of LA-OH and LA treatments on relative water content **(A)** and relative electrolyte leakage **(B)** in leaves under control and drought circumstances are shown in the graphs. The values represent the SD of the mean of three replicates. Duncan’s test and ANOVA were used. Significant differences (P < 0.05) are indicated by different letters.

Pretreatment with 50 ppm LA and LA-OH reduced the withering of peach leaves under drought stress (**Figure 1**). Under drought conditions, the RWC value of peach leaves decreased considerably. However, pretreatment with LA and LA-OH greatly slowed the reduction in water content **(** **Figure 2A**). During drought stress, the REL of untreated peach seedlings increased dramatically, but the REL of plants pretreated with LA increased significantly (**Figure 2B**). These results indicate that under drought conditions, LA and LA-OH enhanced RWC while decreasing REL.

### Characteristics of photosynthetic parameters of leaves

**Figure 3:**
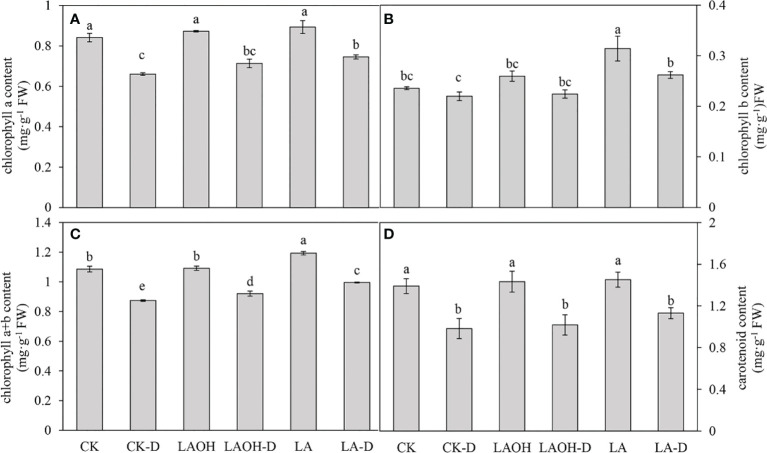
Effects of LA-OH and LA on photosynthetic pigment content in P. persica leaves under drought stress **(A)** chlorophyll a content; **(B)** chlorophyll b content; **(C)** chlorophyll a+b content; **(D)** carotenoid content). Data are mean ± SD (n = 3). Duncan’s test and ANOVA were used. Significant differences (P < 0.05) are indicated by different letters.

CK treatment considerably decreased the net photosynthetic rate (Pn), stomatal conductance (Gs), transpiration rate (Tr), water use efficiency (WUE), and SPAD value under drought stress while dramatically increasing the intercellular CO_2_ concentration (Ci) (**Figure 3**). Exogenous LA or LA-OH substantially slowed the decreases in Pn, Gs, Tr, WUE, and SPAD during drought stress (P<0.05). In comparison to the CK-D subgroup, the LA-D and LA-OH-D treatment subgroups exhibited increases in Pn, Gs, Tr, WUE, and SPAD of 204.55%, 54.82%, 134.14%, 33.13%, 28.09% and 115.91%, 18.76%, 104.87%, 4.47%, 19.03%, respectively. However, compared to the Ci values for the CK-D drought stress treatment subgroup, Ci in the LA and LA-OH drought stress treatment subgroups fell by 15.24% (LA-D) and 13.81% (LA-OH-D), demonstrating that exogenous LA and LA-OH can ameliorate drought stress by influencing the photosynthetic properties of peach leaves. The impact of LA was superior to that of LA-OH.

### The effect of exogenous LA and LA-OH on leaf stomatal characteristics

**Figure 4:**
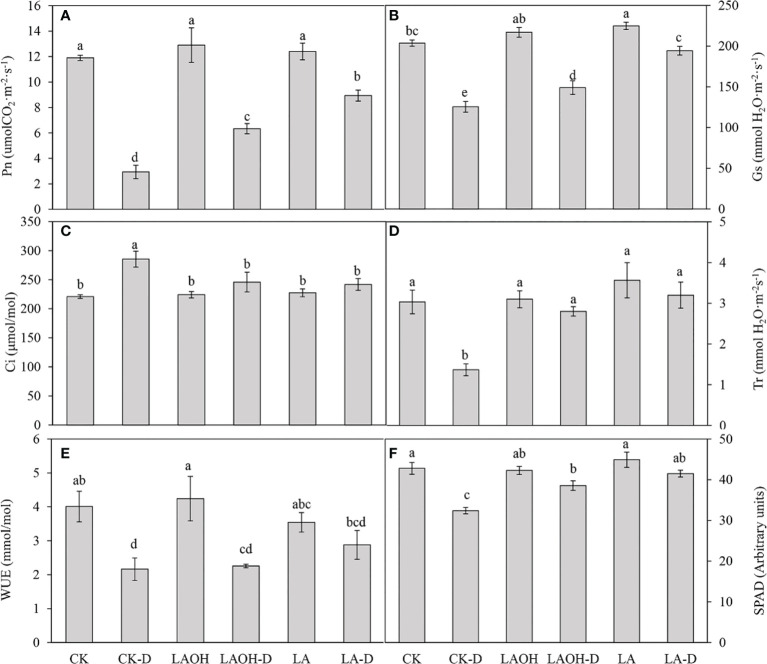
Effects of LA-OH and LA on the sizes of stomatal apertures of P. persica leaves under drought stress **(A)** light microscopeto visualize showed the characteristics of stomata; **(B)** Stomatal aperture area). Data are mean ± SD (n = 3). Duncan’s test and ANOVA were used. Significant differences (P < 0.05) are indicated by different letters.

Under normal conditions, the stomatal opening of the drought-treated leaves was substantially less than that of the control leaves. Exogenous LA-OH and LA may enlarge the stomatal aperture of leaves (**Figure 4A**); compared to the CK-D treatment, the LA-OH-D and LA-D treatments considerably enhanced the average stomatal aperture, increasing it by 2.23 times and 2.41 times, respectively (**Figure 4B**).

### Effects of drought stress on leaf pigmentation

**Figure 5:**
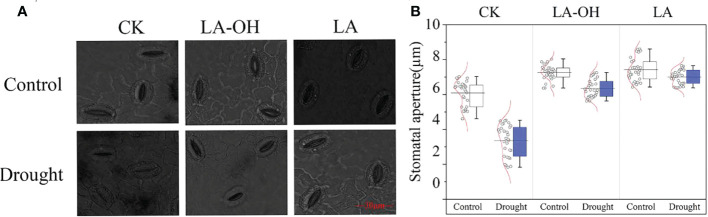
Effects of LA-OH and LA on the photosynthetic characteristics of P. persica under drought stress **(A)** Pn; **(B)** Gs; **(C)** Ci; **(D)** Tr; **(E)** WUE; **(F)** SPAD). Data are mean ± SD (n = 3). Duncan’s test and ANOVA were used. Significant differences (P < 0.05) are indicated by different letters.

Drought stress reduced the concentrations of chlorophyll a, chlorophyll b, chlorophyll a+b, and carotenoids in the peach seedlings by 21.47%, 6.78%, 19.53%, and 29.30%, respectively. By contrast, the concentrations of chlorophyll a, chlorophyll b, chlorophyll a+b, and carotenoids in the LA-OH-D treatment subgroup increased by 8.01%, 2.00%, 5.36%, and 3.53%, respectively (**Figure 5**). In the LA-D treatment subgroup, the concentrations of chlorophyll b, chlorophyll a+b, and carotenoids increased by 12.85%, 19.19%, 13.92%, and 15.06%, respectively. Exogenous LA-OH and LA can stimulate the synthesis of photosynthetic pigments in peach leaves under drought stress, with LA having a greater impact than LA-OH.

### The effects of LA and LA-OH on the osmotic control system of drought-stressed peach seedlings

**Figure 6:**
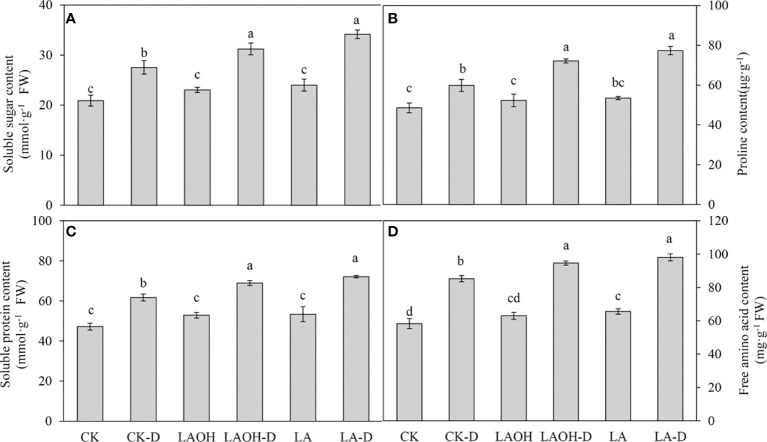
Effects of LA-OH and LA on osmoregulatory substances in *P. persica* under drought stress **(A)** soluble sugar content; **(B)** proline content; **(C)** soluble protein content; **(D)** free amino acid content). Data are mean ± SD (n = 3). Duncan’s test and ANOVA were used. Significant differences (P < 0.05) are indicated by different letters.

Drought stress treatment dramatically enhanced the amounts of soluble sugar, proline, soluble protein, and free amino acids in peach seedlings not treated with LA-OH or LA (**Figure 6**). The levels of soluble sugar, proline, soluble protein, free amino acids, and other organic osmotic regulators were much higher in drought-stressed plants treated with LA-OH and LA than in untreated drought-stressed plants. In contrast with the subgroups not treated with LA-OH or LA, the levels of soluble sugar, proline, soluble protein and free amino acids increased by 13.48%, 20.51%, 11.82% and 10.96%, respectively, in the LA-OH treatment subgroups. Under LA treatment, these levels increased by 24.11%, 29.30%, 16.89% and 15.04%, respectively.

### Changes in chlorophyll fluorescence in LA-OH and LA peach seedlings

**Figure 7:**
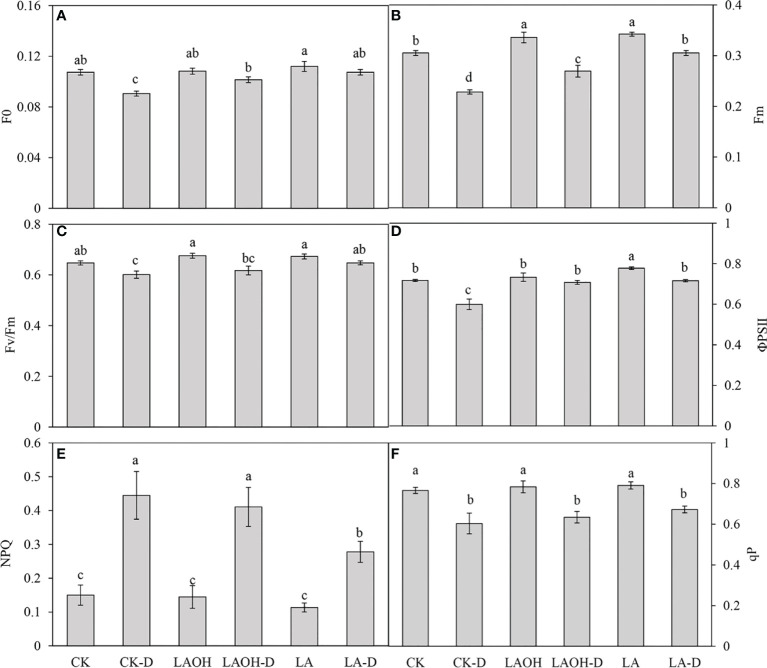
Changes in chlorophyll fluorescence parameters of peach seedings under drought conditions. **(A)** F0; **(B)** Fm; **(C)** Fv/Fm; **(D)** ΦPSII; **(E)** NPQ; **(F)** qP. Duncan’s test and ANOVA were used. Significant differences (P < 0.05) are indicated by different letters.

**Figure 8:**
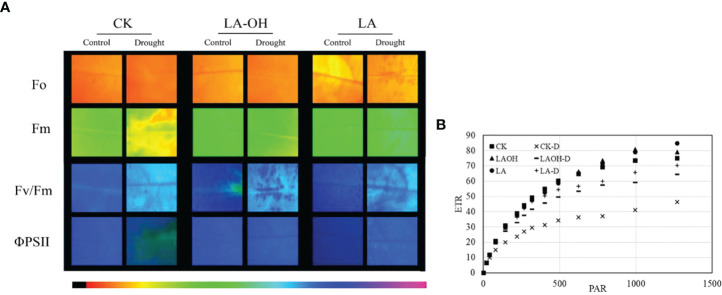
Chlorophyll fluorescence parameters in leaves of the peach seedings. **(A)** Chlorophyll fluo-rescence imaging of F0, Fm, Fv/Fm and ΦPSII; The darker the color, the greater the Fv/Fm; **(B)** ETR-PAR.

In the absence of LA-OH or LA treatment, the initial fluorescence value (F0), maximum fluorescence value (Fm), maximum quantum yield of PSII (Fv/Fm), real photochemical efficiency of ΦPSII (ΦPSII), and photochemical quenching coefficient (qP) of drought-treated peach tree seedlings were considerably reduced (**Figure 7**). LA-OH treatment in the LA-OH-D subgroup enhanced F0, Fm, Fv/Fm, ΦPSII, and qP by 12.03%, 17.95%, 2.7%, 18.38%, and 5.07%, respectively, compared to the values for the CK-D subgroup. The impact of LA treatment was much greater than that of the LA-OH treatment, with LA treatment enhancing F0, Fm, Fv/Fm, ΦPSII, and qP by 18.54%, 33.71%, 7.75%, 19.41%, and 11.39%, respectively. Under drought stress, the nonphotochemical quenching coefficient (NPQ) increased considerably in all treatment groups, with the CK-D treatment subgroup having the highest level, followed by the LA-OH-D treatment subgroup, with no significant difference between the two. Under drought stress, the LA treatment resulted in the lowest NPQ level, which was substantially different from the levels in the CK-D and LA-OH-D subgroups. The corresponding colour photos of leaves show the state of the four parameters under various treatments (**Figure 8A**). LA-OH-D treatment and LA-D treatment effectively alleviated the damage caused by drought stress. The ETR-PAR light response curve (**Figure 8B**) revealed that the LA-OH-D and LA-D treatments greatly increased the ETR_max_ of peach tree seedlings by 38.84% and 51.45%, respectively.

### LA-OH and LA alleviated oxidative damage in peach seedlings under drought stress

**Figure 9:**
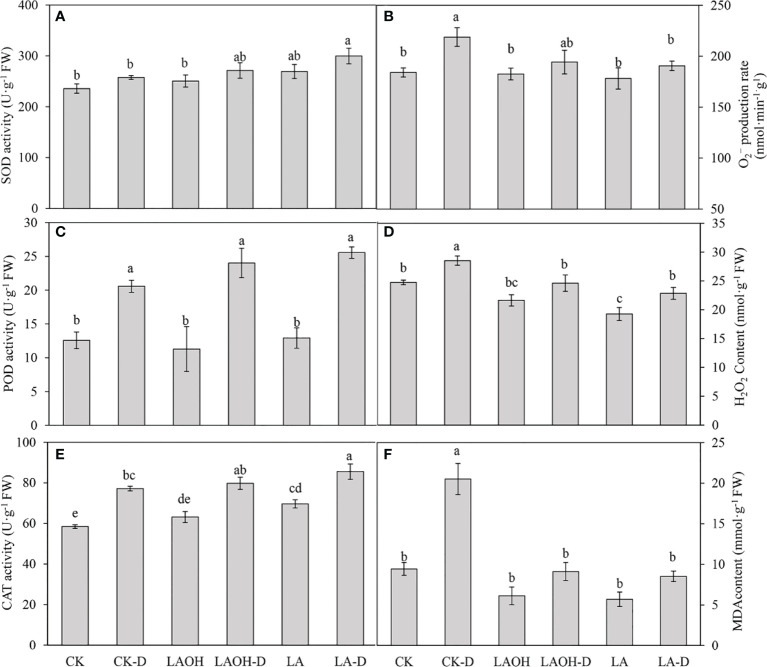
Effects of LA and LA-OH on antioxidant enzyme activity and MDA content of *P. persica* leaves under drought stress **(A)** SOD; **(B)** O_2_^−^production rate; **(C)** POD; **(D)** H_2_O_2_ content; **(E)** CAT; **(F)** MDA content). Data are mean ± SD (n = 3). Duncan’s test and ANOVA were used. Significant differences (P<0.05) are indicated by different letters.

We examined the levels of O_2_^-^ and H_2_O_2_ to investigate the influence of LA-OH and LA pretreatment on oxidative alterations. Under drought conditions, O_2_^-^ and H_2_O_2_ accumulated in peach leaves, and the levels of O_2_^-^ and H_2_O_2_ in peach leaves treated with LA-OH and LA were lower than those in leaves not treated with LA-OH or LA (**Figure 9**), suggesting that LA-OH and LA may mitigate ROS-induced oxidative damage. The SOD, POD, and CAT activity levels of peach leaves under drought stress increased considerably when compared to the activity levels of the control, but the SOD, POD, and CAT activity levels of peach leaves treated with LA-OH and LA were greater than those under drought stress without LA-OH or LA treatment, increasing by 5.37%, 16.74%, 3.36%, and 16.38%, 24.25%, 10.77%, respectively. Furthermore, the antioxidant enzyme activity in the LA-D treatment subgroup was greater than that of the LA-OH-D treatment subgroup. Drought stress, on the other hand, considerably enhanced the MDA content in peach leaves when compared to the MDA content in the leaves of the control. The MDA content in peach leaves treated with LA-OH and LA was substantially lower than that in leaves under drought stress without LA-OH or LA treatment but was significantly higher than that of the control.

### Correlation analysis of LA-OH and LA with drought resistance

**Figure 10:**
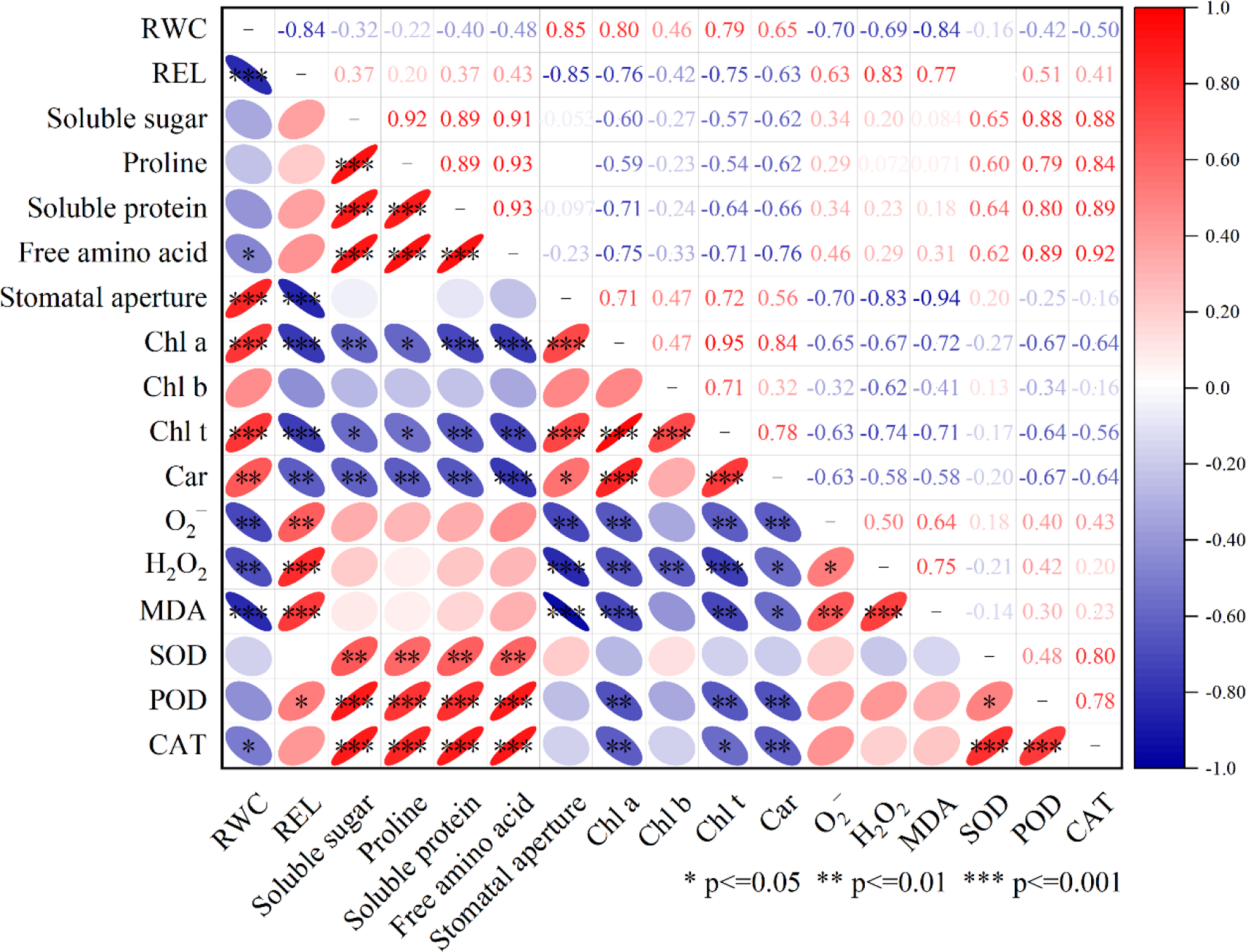
Pearson correlation analysis was done on RWC, REL, Soluble sugar, Proline, Suloble protein, Free amino acid, Stomatal aperture, Chl a, Chl b, Chl t, Car, O_2_^-^, H_2_O_2_ MDA, SOD, POD, CAT after LA-OH and LA treatments under drought stress. ***—indicates 0.1% significant (p≤ 0.001), **—indicates 1% significant (p≤ 0.01), and *—indicates 5% significant (p≤ 0.05).

For the correlation study, the relative water content (RWC) of the peach leaves, as well as leaf pigment, osmotic regulatory system, stomatal aperture, and antioxidant system activity (i.e., O_2_^-^, H_2_O_2_, MDA, SOD, POD, CAT), were evaluated (**Figure 10**). The RWC of leaves was found to be positively correlated with stomatal aperture and leaf pigment content and was significantly correlated with stomatal aperture (*r*=0.85), significantly positively correlated with chlorophyll a (Chl a) (*r*=0.80), positively correlated with chlorophyll b (Chl b) (*r*=0.46), significantly positively correlated with total chlorophyll (Chl t) (*r*=0.79), and significantly positively correlated with carotenoid (Car) (*r*=0.65). RWC was significantly negatively correlated with REL (*r*=-0.84); negatively correlated with soluble sugar (*r*=-0.32), proline (*r*=-0.22), and soluble protein (*r*=-0.40); and significantly negatively correlated with free amino acids (*r*=-0.48). RWC was also significantly negatively correlated with O_2_^-^ (*r*=-0.70), H_2_O_2_ (*r*=-0.69) and MDA (*r*=-0.84); negatively correlated with SOD (*r*=-0.16) and POD (*r*=-0.45); and significantly negatively correlated with CAT (*r*=-0.50).

## Discussion

Fatty acids (FAs) are ubiquitous in nature and play an important role in many biological processes (Liu et al., 2008). FAs are significant physiological molecules that play a role in cellular energy storage, membrane structure, and numerous signalling pathways (Corino et al., 2004 **;** Shin et al., 2007). FAs are well recognized for their antibacterial and antifungal properties. (Agoramoorthy et al., 2007). Lauric acid (LA) is one of the most active medium-chain FAs (Dayrit, 2015), and its antimicrobial benefits in medicine (Carballeira, 2008 **;** Liang et al., 2021) and plants have been described (Řiháková et al., 2001; Walters et al., 2003), although there have been few investigations on its role in boosting plant drought tolerance. LA not only promotes the *in vitro* growth of zygotic coconut (*Cocos nucifera* L.) embryos, but plants treated with LA in the predomestication stage can be used to improve crop drought resistance because of the role of LA in modulating the degree of stomatal opening and closing, maintaining the relative moisture content in leaves, and maintaining photosynthetic activity (Medeiros et al., 2015). Studies have shown that drought stress has a significant effect on loquat growth and metabolism and that among leaf metabolites, LA is involved in metabolism in loquat under drought conditions (Gugliuzza et al., 2020). According to research, the products of fatty acid oxidase in organisms appear to have vital biological actions (Funk, 2001 **;** Blée, 2002). Moreover, it is now known, that they are engaged in a variety of biological activities. Different aspects of plant growth, as well as responses to biotic and abiotic stressors, entail oxidative products (Kandel et al., 2006 **;** Nelson, 2006). In plants, 12-hydroxylauric acid (LA-OH) is mostly generated through a process mediated by cytochrome P450 fatty acid hydroxylases. Research indicates that the addition of hydroxylauric acid has a role in regulating plant development phenomena (Imaishi and Petkova-Andonova, 2007). It has been shown that LA and LA-OH activate the immune response in Arabidopsis by activating cytosolic calcium ([Ca2+]_cyt_) signalling (Kutschera et al., 2019). We first investigated the impacts of exogenous LA and LA-OH on plant photosynthetic performance under drought conditions, and we then investigated the mechanism of LA and LA-OH in photosynthesis by evaluating photosynthetic pigments, gas exchange parameters, and chlorophyll fluorescence parameters. We describe our findings by examining osmoregulation and antioxidant systems because the use of LA and LA-OH under abiotic stress has not been adequately studied.

The ability of plants to respond to drought stress is enhanced by maintaining the correct leaf water status (Han et al., 2022). Drought decreased the relative water content (RWC) of peach leaves, and after irrigating the seedlings with LA and LA-OH, RWC in subgroups receiving one of these two treatments was considerably higher than RCW in the CK-D subgroup (**Figure 2**). Morphological observation of the seedling leaves revealed that the more severe the drought was, the more severe the water loss in the leaves, and the greater the degree of curling and folding of the leaves. Relative electrolytic leakage (REL) is a good predictor of membrane permeability and drought tolerance (Sun et al., 2018). Our findings revealed that the REL value of the CK-D treatment was greatly enhanced; however, the REL values of the LA-D and LA-OH-D treatment subgroups were not significantly different from those of the subgroups receiving LA or LA-OH treatments under drought conditions. The difference was not statistically significant, suggesting that LA and LA-OH treatment prevented cell damage under dry conditions.

Photosynthesis, the process through which plants obtain carbon and energy, is particularly susceptible to environmental stress (Muhammad et al., 2021). Research indicates that drought stress can disrupt pigment complexes, impede electron transport, damage chloroplast structure, and reduce photosynthetic rates (Liu et al., 2016 **;** Li et al., 2018 **)**. Pn can be decreased under drought stress due to decreased chlorophyll content and stomatal conductance (Liang et al., 2018). In our study, drought reduced chlorophyll a, chlorophyll b, and carotenoid levels; however, the application of LA-OH and LA mitigated the deleterious effects of drought on chlorophyll content. Our findings agree with those of Moaveni (2011), who discovered that photosynthetic pigments are decreased in crops subjected to water stress. Drought stress reduces stomatal aperture, which results in a decrease in the Pn rate or in alterations to photosynthetic metabolism. Furthermore, drought decreases the CO_2_ concentration and transpiration rate *via* stomata (Moaveni, 2011; Shahid et al.,2014). Our research revealed that LA-OH and LA treatments might stimulate stomatal opening in peach leaves while also significantly reducing stomatal aperture closure caused by drought. Our findings imply that LA-OH and LA increase plant drought tolerance through a variety of mechanisms, including by decreasing stomatal limitation, protecting chlorophyll from degradation, and improving photosynthetic capability.

Evaluation of chlorophyll fluorescence characteristics is an essential technique for determining plant water status during drought stress because these characteristics can rapidly, precisely, and safely indicate the effects of drought stress on plant photosynthesis (Maxwell and Johnson, 2000). F0 is a key metric for assessing plant stress damage, and Fm can indicate PS II electron transport capabilities following dark adaptation (Rao et al., 2021). Our investigation revealed that during drought stress, both F0 and Fm declined dramatically but that LA-OH and LA treatments could successfully delay the declining trend (**Figure 8**). Fv/Fm is an excellent measure for determining the degree of damage to plant leaves, and Fv/Fm decreases with environmental stress (Pettigrew, 2004 **;** Raja et al., 2020). Under drought stress, all three drought treatment subgroups (CK-D, LA-OD-D, and LA-D) exhibited a decline in Fv/Fm. Among the subgroups, the CK-D subgroup exhibited a markedly decreased Fv/Fm, while the LA-OH-D and LA-D subgroups exhibited the smallest decrease, showing that drought stress had a markedly impact on the seedlings in the CK treatment subgroup. The PS II response centre was damaged. As a result, its activity and light energy conversion efficiency were reduced. The seedlings in the LA-OH-D and LA-D subgroups, on the other hand, incurred less damage and showed marked tolerance to dry environments. During drought stress, the maximum quantum efficiency (Fv/Fm) of photosystem PS II, the actual quantum efficiency of PS II (Φ PSII), and the photochemical quenching (qP) all declined, although the nonphotochemical quenching (NPQ) increased (**Figures 7** **C, D**). Gao et al. (2021) reported comparable results in apple. In our study, the reduction in these parameters was somewhat reversed after applying LA-OH and LA. The LA-OH and LA treatments not only reduced the drought-induced decreases in Fv/Fm and ΦPSII but also promoted restoration of photosynthesis. Simultaneously, damage to a key component of the PS II response centre was minimized. The perceived electron transfer efficiency under real light intensity is represented by the photosynthetic electron transfer rate (ETR). Under normal conditions, this value reflects the electron capture efficiency of the PS II reaction centre. Plant ETR values are normally steady within a particular range, and their ETR decreases when they are stressed (Rao et al., 2021). In this study, irrigation with LA-OH and LA under drought conditions significantly increased leaf ETR_max_, indicating that improving leaf light energy use efficiency had a significant promoting effect, whereas the greatest decrease in leaf ETR_max_ was observed in the CK-D treatment subgroup, indicating that electron transport was severely hampered, in turn affecting photosynthetic capacity (**Figure 9**).

Plant resistance to abiotic stressors is heavily reliant on the accumulation of osmoprotectants (Jespersen et al., 2017). LA-OH and LA had substantial impacts on various osmolytes in our investigation. Plants under drought stress had higher levels of soluble sugar and soluble protein than those without LA-OH or LA treatment (**Figure 6**). The levels of proline and free amino acids in the plant are major regulators of cellular osmotic potential. The build-up of these biomolecules aids in cell membrane integrity and the prevention of osmotic and oxidative damage (Han et al., 2022). LA-OH and LA treatments have significantly boosted proline content in drought-stressed pecan seedlings in our past trial studies. Comparable results have been obtained in apple (Gao et al., 2021). In addition to its osmoregulatory role, proline accumulation under harsh conditions protects the photosynthetic system from damage (Khan et al., 2016).

It is critical for cells to maintain reactive oxygen species equilibrium and osmotic potential during drought (Wang et al., 2013). In our study, drought stress caused an excessive concentration of ROS in leaves, disrupting the plant cell membrane system and causing electrolyte leakage, which resulted in chlorophyll degradation. These modifications have been demonstrated to significantly limit photochemical processes, impair photosynthesis, and hasten leaf senescence (Gao et al., 2020). In the current investigation, the levels of O_2_^-^, H_2_O_2_, and MDA in peach leaves increased dramatically under drought stress but decreased significantly following the application of LA-OH or LA (**Figure 9**). The antioxidant defence system in plants, which comprises enzymatic and nonenzymatic antioxidants, strictly controls the equilibrium of reactive oxygen species (Wang et al., 2013). According to studies, the external application of acetic acid can control reactive oxygen species homeostasis by increasing the activity of apple antioxidant enzymes, which guards the fruit against harm caused by reactive oxygen species during drought stress (Sun et al., 2022). Trehalose treatment has significantly increased the activity levels of SOD, CAT and POD enzymes in sunflower leaves under water stress, thus upregulating the oxidative defence system of plants, which has a considerable effect on promoting plant growth under drought stress conditions (Kosar et al., 2021). The LA-OH or LA treatment in our study greatly boosted the activity levels of SOD, POD, and CAT, according to our findings (**Figure 9**). Excess reactive oxygen species have been found to be eliminated by SOD, POD, and CAT enzymes in other studies. SOD can turn O_2_^-^ into H_2_O_2_, and H_2_O_2_ can subsequently be transformed back into H_2_O through POD and CAT to reduce reactive oxygen species damage. Increasing the activity of enzymatic reactive oxygen species scavengers and the quantity of nonenzymatic reactive oxygen species scavengers in grape has been shown to be beneficial (Wang and Li, 2006). We came to the same conclusion for peach seedlings.

The interplay of these indicators was investigated using Pearson’s correlation analysis to assess how the use of LA-OH and LA increase plant tolerance under drought conditions. The correlation analysis in our study revealed that ROS and MDA were positively correlated with enzymatic antioxidants, while RWC was positively correlated with ROS, REL, reactive oxygen species, the osmotic regulation system, and the enzymatic antioxidant system (**Figure 10**). The results demonstrated that drought stress had a significant impact on the photosynthetic system, osmotic regulatory system, and reactive oxygen species enzymatic antioxidant system of peach seedlings and that LA-OH and LA could mitigate these effects to alleviate drought stress.

## Conclusion

In this study, both LA-OH and LA alleviated the growth of peach seedlings under drought stress. Drought stress can be alleviated *via* various mechanisms, including the following: (i) LA-OH and LA can effectively increase the chlorophyll content in peach leaves, increase the degree of leaf stomatal opening and enhance the net photosynthetic rate, thereby improving photosynthesis and reducing the damage caused by drought stress to the photosystem; (ii) LA-OH and LA can increase the contents of soluble sugar, soluble protein, proline, and free amino acids; alleviate osmotic stress; control the relative conductivity of leaves; and maintain the leaf water content; (iii) under drought stress, both LA-OH and LA may decrease the O_2_^-^ and H_2_O_2_ contents and lipid peroxidation level in tobacco plants, which is beneficial for the antioxidant system of peach seedlings. In general, both LA-OH and LA can help to decrease drought stress. The findings of this research shed light on the mechanism by which LA-OH and LA relieve drought stress in peach plants. Additional research is needed to better understand these pathways.

## Data availability statement

The raw data supporting the conclusions of this article will be made available by the authors, without undue reservation.

## Author contributions

BZ: substantial contributions to the conception or design of the work; or the acquisition, analysis, or interpretation of data for the work; HD: substantial contributions to the acquisition, analysis; MS: drafting the work or revising it critically for important intellectual content; XW and ZW: substantial contributions to the interpretation of data for the work; YX: provide approval for publication of the content; FP: agree to be accountable for all aspects of the work in ensuring that questions related to the accuracy or integrity of any part of the work are appropriately investigated and resolved. All authors contributed to the article and approved the submitted version.

## Funding

This work was supported by the National Key Research and Development Program of China (2020YFD1000203), the National Modern Agro-industry Technology Research System Fund (Grant No. CARS-30-2-02).

## Conflict of interest

The authors declare that the research was conducted in the absence of any commercial or financial relationships that could be construed as a potential conflict of interest.

## Publisher’s note

All claims expressed in this article are solely those of the authors and do not necessarily represent those of their affiliated organizations, or those of the publisher, the editors and the reviewers. Any product that may be evaluated in this article, or claim that may be made by its manufacturer, is not guaranteed or endorsed by the publisher.
